# Sinonasal Cancer: Improving Classification, Stratification and Therapeutic Options

**DOI:** 10.3390/cancers15061675

**Published:** 2023-03-09

**Authors:** Mario A. Hermsen, Paolo Bossi, Alessandro Franchi, Matt Lechner

**Affiliations:** 1 Department Head and Neck Oncology, Instituto de Investigación Sanitaria del Principado de Asturias, 33011 Oviedo, Spain; 2 Head and Neck Medical Oncology Department, Fondazione IRCCS Istituto Nazionale dei Tumori, 20133 Milan, Italy; 3 Department of Translational Research and of New Technologies in Medicine and Surgery, University of Pisa, 56126 Pisa, Italy; 4 UCL Cancer Institute, University College London, London WC1E 6BT, UK; 5 Division of Surgery and Interventional Science, Academic Head and Neck Centre University College London, London WC1E 6BT, UK

The nasal cavities and paranasal sinuses are the site of origin of a wide spectrum of histologically and clinically distinct disease entities [1,2]. The classification of tumors arising from these sites can be challenging when using routine diagnostic markers, particularly as there are many poorly differentiated subtypes with overlapping features. Our Special Issue on sinonasal cancer includes novel and highly exciting findings on sinonasal squamous cell carcinoma (SNSCC), inverted papilloma (ISP), intestinal-type adenocarcinoma (ITAC), undifferentiated carcinoma (SNUC), neuroendocrine carcinoma (SNEC), olfactory neuroblastoma (ONB), SMARCB1- and SMARCA4-deficient sinonasal carcinomas, and malignant mucosal melanoma (MMM). 

In spite of some therapeutic advances in the field of head and neck cancer management, recurrences remain frequent for some subtypes and the overall 5-year survival rate is still low (20–70%), with a huge variability according to histology and stage [3,4]. This poor prognosis is likely multifactorial and may be caused by intrinsic tumor factors, such as subtype and resistance to irradiation and systemic therapy as well as the difficulty to achieve complete resection due to the extent of the tumor or the proximity of important anatomical structures, such as the orbit and brain. With a combined incidence of approximately 0.5–1.0 cases per 100,000 per year, sinonasal malignancies are considered rare cancers [5]. Although rare tumors comprise approximately 20% of all cancer patients, new advances lag significantly behind to those reported in more common solid cancers [6]. Comprehensive molecular studies are scarce and very few patients with rare cancers are offered enrollment in clinical trials. Nevertheless, some progress has been made in recent years with regard to surgical techniques, precision imaging modalities and radiotherapy, and in the identification of molecular alterations that may improve diagnosis, prognosis and the stratification of treatment.

Indeed, the upcoming WHO Classification of Head and Neck Tumors already recognizes a number of tumor entities based on protein expression, chromosomal translocations, oncogenic virus infection or specific gene mutations [1,2]. Examples are NUT carcinoma defined by t(15;19) NUT-BRD4 rearrangement and SMARCB1 (INI-1)- and SMARCA4 (BRG1)-deficient sinonasal carcinomas characterized by the absence of SMARCB1 or SMARCA4 protein expression. A detailed description of diagnostically relevant genetic aberrations can be found in the paper by Taverna et al. in this Special Issue [7]. In addition, methylation profiling has been shown to allow the classification of sinonasal tumor subtypes, even identifying subgroups of tumors within SNUC and SNEC [8]. A similar approach to aid and finetune the diagnosis of the multitudinous subtypes of brain tumors has now been implemented by the WHO Classification of Tumors [9]. Further confirmatory studies will be needed to reach such a consensus for the classification of sinonasal tumors by contrasting methylation profiling and testing for other common molecular alterations with histopathological examination. 

Genetic analysis also allows for the identification of molecular targets for modern targeted therapies. Several studies on tumor-infiltrating lymphocytes and aspects of the PD-L1/PD1 checkpoint have indicated a role for immunotherapy in several sinonasal tumor subtypes [10,11,12,13,14,15,16], and we strongly encourage research into the clinical response to treatment with immune checkpoint inhibitors in sinonasal cancers such as recently reported on MMM [17]. Next-generation sequencing identified recurrent subtype-unique mutations including: *APC*, *CTNNB1*, *PIK3CA* and *KRAS* in ITAC, *EGFR* and *CDKN2A* in SNSCC, *NRAS* and *NF1* in MMM, *IDH2* in SNUC, and *ARID1A*, *SMARCB1* and *SMARCA4* in SNEC, SNUC and TCS [18,19,20,21,22,23,24,25,26]. Other markers such as Somatostatin receptor 2 (SSTR2) expression were also reported in sinonasal cancers, such as in ONB [27]. Moreover, genetically well-characterized stable tumor cell lines, organoids and animal models are becoming available for the preclinical testing of candidate therapeutic agents [28,29,30,31,32]. In summary, these studies may form the basis for a personalized treatment approach for sinonasal cancers. Candidate therapies include PI3K/mTOR inhibitors for ITAC, EGFR and CDK4/6 inhibitors for SNSCC, MEK inhibitors for MMM, IDH2 inhibitors for SNUC/SNEC/ONB, and EZH2 inhibitors for SNUC/TCS, while DNA repair and FGFR inhibitors may be considered for many of the sinonasal tumor subtypes.

This Special Issue contains 15 papers on genetic, histopathological and clinical aspects of sinonasal tumors, including eight studies presenting new data on genetic aberrations, two studies on etiology and molecular pathology and five reports on advances in surgical approaches and clinical management.

In an extensive study of 220 sinonasal tumors and 10 cell lines, Hieggelke et al. reported that 3% of primary SNSCC and one SNSCC cell line (SCCNC5) display mismatch repair deficiency and 5% of high-risk and 10% of low-risk HPV. Using an NGS panel of 36 cancer-related genes, they reported recurrent *EGFR* exon 20 mutations in ISP (89%) and in ISP-related SNSCC (74%), but none in other sinonasal tumor types [33]. Focusing on these mutations and investigating their candidacy as targets for therapy with modern inhibitors, Pacini et al. present a detailed overview of what is known on *EGFR* exon 20 mutations in non-small cell lung cancer (NSCLC) and in SNSCC and the lessons that can be learned from the targeted treatment of NSCLC for application to SNSCC [34]. Other mutations reported by Hieggelke et al. included *TP53*, *PIK3CA*, *CDKN2A* and were commonly seen in SNSCC, SNEC, SNUC and SNAC. The authors conclude that testing of genetic biomarkers including mismatch repair testing will provide useful information for an individualized therapeutic strategy [33]. Results of a similar NGS approaches were presented in two papers focusing on poorly differentiated sinonasal tumors and on ITAC [20,35].

Libera et al. combined NGS using a 22 cancer-gene panel with additional immunohistochemistry, PCR sequencing, MLPA gene copy number and LINE-1 methylation analysis on 53 cases of SNUC, SNEC and SNSCC [35]. SMARCB1 deficiency and *IDH2* mutation was found in nine and five cases, thus changing the original diagnosis of these tumors. They also showed that these 14 cases had significantly higher LINE-1 methylation levels and that this change was associated with a poorer clinical outcome. They also reported that *IDH2*-mutated tumors are associated with a worse outcome, which is different from what had been published in the literature beforehand [25,36] and larger studies are required to confirm these findings. Having said this, their reported association of *IDH2*-mutation as well as SMARCB1 deficiency with aberrant global methylation was later confirmed by a very recent large methylation study on sinonasal cancers [8].

Riobello et al. applied a 120-gene NGS panel to a cohort of 50 ITAC and reported on four signaling pathways that appear to be frequently affected in ITAC by mutations, specifically the Wnt, DNA repair, MAPK and PI3K pathway, in 20–30% of cases. Neither were these mutations mutually exclusive nor were they specific for histologically diagnosed subtypes of ITAC and they were not significantly associated with clinical outcomes or other clinical variables. The authors conclude that ITAC is genetically heterogeneous and that the four signaling pathways may be amenable to therapeutically targeting this disease [20]. Another interesting study on ITAC was published by Schatz et al. They studied 145 cases of ITAC and demonstrated significant upregulation of several eukaryotic translation initiation factors downstream of MAPK and PI3K signaling pathways. These may represent novel candidate therapeutic targets [37].

The high incidence of recurrences and distant failures in MMM is the main cause of death, with reported one-, three-, and five-year recurrence-free and overall survival of 61, 31, and 22%, and 78, 49, and 38%, respectively [17]. With the aim to tackle this, Freiberger et al. presented a pioneer longitudinal study on MMM describing an observed ‘switch’ from *KRAS*, *KIT* or no mutation in primary tumors to solely genomic alterations in *NRAS* in recurrent tumor, after developing resistance to immunotherapy. Their exciting results provide a rationale for combined treatment with MEK and PD-1/PD-L1 immune checkpoint inhibitors in MMM [38].

Two studies have shed more light on the role of HPV in SNSCC [39,40]. Both studies found that HPV-positive SNSCC patients have a more favorable survival than HPV-negative cases, similar to what had been described for oropharyngeal squamous cell carcinoma [41]. Both also agree that p16 immunohistochemistry is not a reliable surrogate marker for HPV, in line with previous results showing that p16 IHC is not reliable as a marker outside the oropharynx [42] and that HPV E6/E7 mRNA expression may be the gold standard for the determination of HPV status. However, p16 may still play a role in the carcinogenesis of this disease and the loss of expression, as well as *CDKN2A* inactivating mutations, and may play a role in the progression from ISP to SNSCC [19,43].

Taverna et al. provide a comprehensive overview of the histology and molecular pathology of sinonasal tumor subtypes in general. They described the latest diagnostically relevant findings of a field that progresses quickly [7]. Specifically focusing on adenocarcinomas, Leivo et al. demonstrate geographical differences in the incidence of ITAC and non-intestinal-type adenocarcinoma, possibly reflecting differences in wood dust exposure as an etiological factor [44].

Finally, five papers in this Special Issue give an excellent overview of advances in imaging, surgery and radiotherapy techniques, their application and effectiveness for sinonasal tumors, and the challenges that still lie ahead. Salfrant et al. present a detailed description of pre-surgery imaging with regard to tumor extension and invasion into the orbit and brain, which is relevant for prognostic evaluation and treatment planning [45]. Chatelet et al. provide a complete overview of surgical techniques, the criteria to choose which ones to apply to sinonasal tumors to and the clinical outcomes and morbidities of the different techniques [46]. Gallioni et al. focused on the challenges with managing possible lymph node metastases in the neck region, whose incidence differs among the sinonasal cancer subtypes and increases during follow-up. Choices for a watchful waiting approach or surgical or radiotherapeutic elective neck treatment are reviewed [47]. Thariat et al. describe developments in radiotherapy, including precision intensity-modulated radiotherapy and charged particle therapy, techniques that require skill and good knowledge of the anatomy of the sinonasal cavities that can change during the course of radiotherapy. Choices for types of radiotherapy in relation to better local control and decrease toxicity are discussed [48]. Finally, Eide et al. show how all these clinical advances, but also molecular pathological aspects are to be taken into consideration specifically for the treatment of ISP and associated SNSCC, and how they may affect clinical outcome [49].

The papers published in this Special Issue demonstrate the immense value of multidisciplinary international collaboration between ENT physicians, pathologists, radiotherapists, oncologists and geneticists to advance clinical management and outcomes. In the coming years, further progress could be made if patients with these very rare cancers are treated in designated centers of excellence that can accumulate the necessary clinical expertise. Telepathology networks could be set up to discuss difficult-to-diagnose cases and specialized centers could offer affordable molecular genetic analysis, thus standardizing both the classification and identification of clinically actionable alterations for modern therapies.

New initiatives are being taken forward to stimulate such multidisciplinary, multinational research collaboration, e.g., the European Network for Sinonasal Cancer Research (EUSICA) and the European reference network for rare adult solid cancers (EURACAN) in Europe and the Cole-Reagins Registry for Sinonasal Cancer (CORSICA) and Rare Cancer research Program in the USA [50,51,52,53]. The aim of these collaborations is to harmonize the prospective collection of clinical data for comprehensive analysis of multidimensional data, and to share in vitro and in vivo preclinical models, and to provide the ideal set-up to offer patients participation in the latest clinical trials, examples of which are the recently funded proton-beam-therapy PROTIS trial on sinonasal cancer in the UK and the bintrafusp alfa BARON trial on ONB in the USA [54,55].

We, as guest editors, would like to thank all the contributing authors for sharing their excellent work in this Special Issue and the *Cancers* Editorial and Administration team for their technical support. We hope that this Special Issue will encourage and inspire many of our colleagues researching sinonasal cancers and further advance the field. We also hope that we have inspired colleagues to join the various initiatives and for these to prosper and eventually significantly improve clinical care and survival of sinonasal cancer patients across the globe.
